# TGF-β-induced NKILA inhibits ESCC cell migration and invasion through NF-κB/MMP14 signaling

**DOI:** 10.1007/s00109-018-1621-1

**Published:** 2018-01-29

**Authors:** Zhiliang Lu, Zhaoli Chen, Yuan Li, Jingnan Wang, Zhirong Zhang, Yun Che, Jianbing Huang, Shouguo Sun, Shuangshuang Mao, Yuanyuan Lei, Yibo Gao, Jie He

**Affiliations:** 1 Department of Thoracic Surgery, National Cancer Center/Cancer Hospital, Chinese Academy of Medical Sciences and Peking Union Medical College, No. 17 Panjiayuannanli, Beijing, 100021 People’s Republic of China; 2 Thoracic Surgery Department, Beijing Chao-Yang Hospital, Capital University of Medical Science, Beijing, 100020 China

**Keywords:** NF-kappaB-interacting lncRNA, Transforming growth factor β, Long noncoding RNA, Tumor metastasis, ESCC

## Abstract

**Abstract:**

The transforming growth factor β (TGF-β) signaling pathway plays anti- and pro-tumoral roles in the vast majority of cancers, and long noncoding RNAs have been reported to play key roles in the highly contextual response process. However, the roles of long noncoding RNAs (lncRNAs) in TGF-β signaling in esophageal squamous cell carcinoma (ESCC) remain unknown. In this study, we performed RNA-seq to compare lncRNAs expression levels between TGF-β1-treated and untreated ESCC cells and observed that NF-kappaB-interacting lncRNA (NKILA) was remarkably upregulated by the classical TGF-β signaling pathway. RNA profiling of 39 pairs ESCC tumor and adjacent nontumor samples using RT-qPCR demonstrated that NKILA is significantly downregulated in ESCC tumor tissues, and NKILA expression levels were significantly decreased in advanced tumor tissues (III and IV) compared to early stages (I and II) (*p* < 0.01). Gain- and loss-of-function assays showed that NKILA inhibited ESCC cell metastasis in vitro and in vivo, and mechanism studies showed that NKILA repressed MMP14 expression by inhibiting IκBα phosphorylation and NF-κB activation. Collectively, these findings suggest that the TGF-β-induced lncRNA NKILA has potential as an antimetastasis therapy.

**Key messages:**

- Long noncoding RNA NKILA could be remarkably upregulated by classical TGF-β signal pathway in ESCC.
- NKILA was significantly downregulated in esophageal squamous cell carcinoma and negatively correlated with TNM stage.
- NKILA inhibits ESCC cell metastasis via repressing MMP14 expression by suppressing the phosphorylation of IκBα and NF-κB activation.

**Electronic supplementary material:**

The online version of this article (10.1007/s00109-018-1621-1) contains supplementary material, which is available to authorized users.

## Introduction

Esophageal cancer is one of the leading causes of cancer-related death worldwide, and half of the cases of the disease occur in China [1]. Approximately 90% of the cases of esophageal cancer occurring in China and worldwide are squamous cell carcinomas [2–4]. The overall 5-year survival rate of the disease ranges from only 15 to 25%, and the poor outcomes of esophageal cancer are related to its diagnosis at metastatic stages in affected patients, even in cases in which the tumors are superficial [5]. The molecular mechanisms that mediate the metastatic cascade remain largely unknown. Improving the understanding of the molecular mechanisms underlying cancer progression and metastasis may accelerate the development of effective metastasis-targeting therapies for patients with esophageal squamous cell carcinoma (ESCC).

The multifunctional cytokine TGF-β orchestrates an intricate and context-dependent signaling network to modulate tumorigenesis and disease progression. The TGF-β pathway promotes cell cycle arrest and apoptosis in early stage tumors but promotes tumor progression and metastasis in advanced disease, either by inactivating core components of the pathway, such as TGF-β receptors, or by inducing downstream alterations that disable only the tumor-suppressive arm of the pathway [6–8]. The role of TGF-β-induced epithelial-mesenchymal transition (EMT) in cancer cell metastasis has been well-established; however, the necessity of EMT and its opposing process, mesenchymal-epithelial transition (MET), in tumor cell metastasis is still hotly debated [9–11]. A better understanding of the specific and time-dependent downstream effectors of TGF-β signaling is urgently needed to accelerate the development of targeted TGF-β signaling pathway therapies.

Long noncoding RNAs (lncRNAs) are a class of transcripts longer than 200 nucleotides that do not appear to have coding potential [12, 13]. Many studies recently showed that lncRNAs are frequently deregulated in a range of cancers and participate in several biological processes, such as cell cycle progression, cell apoptosis, and cell metastasis [14, 15]. Thus far, the exact mechanisms through which lncRNAs perform their functions remain unknown; however, most lncRNAs appear to perform their bio-functions by regulating vital signaling pathways in cancer, such as the TGF-β signaling pathway, one of the most important cancer cell signaling pathways [15]. For example, the lncRNA-ATB (a lncRNA activated by TGF-β) promoted invasion–metastasis cascade activity and organ colonization by competitively binding with miR-200 and stabilizing IL-11 mRNA [16]. In this study, we found that NF-kappaB-interacting lncRNA (NKILA) was remarkably upregulated by TGF-β1 and focused on the role of NKILA in TGF-β signaling and the invasion–metastasis cascade.

## Materials and methods

### Tissue samples and clinical data collection

A cohort of 39 ESCC tissues and pair-matched adjacent normal esophageal tissues (collected postoperatively from April 2008 to June 2009) were obtained from the Department of Thoracic Surgery of the Cancer Hospital of the Chinese Academy of Medical Sciences. The pathological diagnoses for these 39 patients were obtained. None of patients received radiotherapy or chemotherapy before surgery, nor were any of them diagnosed with any other cancers within 3 years before surgery. The patients with ESCC were staged according to the TNM staging system (the 7th edition) of the American Joint Committee on Cancer, and data pertaining to the clinicopathological characteristics of these patients were collected. The tumor tissues and adjacent normal tissues from these 39 patients were snap-frozen in liquid nitrogen immediately after resection and were stored at −80 °C until used in this study.

### Cell culture and stable cell line construction

A total of seven cell lines, including six ESCC cell lines (KYSE30, KYSE70, KYSE150, KYSE180, KYSE450, and KYSE510) and the immortalized normal human esophageal epithelial cell line Het-1a, were used in this study. The cell lines were cultured as described previously [17]. We confirmed cell line identities by matching the short-tandem repeat (STR) profile of each line to the registered information in the DSMZ online STR database. TNF-α and IL-1β were purchased from Peprotech (USA) and used at a concentration of 10 ng/ml, and TGF-β1 was purchased from R&D (USA) and used at a concentration of 5 ng/ml. The treatment time was 24 h unless otherwise specified. To inhibit TGF-β and NF-κB signaling, we added 10 μM JSH-23 (Selleck, USA) or 5 μM SB505124 (Selleck, USA) to the cell culture media 30 min prior to the specified treatments.

Full-length NKILA cDNA was compounded by Generay (Shanghai, China) and ligated into a pCDH-CMV-MCS-EF1-GFP + Puro (CD513B-1) vector. Non-targeting control shRNA and two shRNAs against NKILA (sh1 and sh2, respectively) were obtained from OBiO (Shanghai, China). The transfection and lentivirus packaging were performed as described previously [17].

### Next-generation sequencing of the transcriptome

KYSE30 and KYSE180 cells were treated with or without 5 ng/ml recombinant TGF-β1 for 72 h. Total RNA was then extracted from the treated and untreated KYSE30 and KYSE180 cells. For the details of sample preparation, library preparation, and sequencing, see the supplementary material. Gene expression levels were determined based on FPKM (fragments per kilobase per million reads) values. We used a fold change ≥ 2.0 as our threshold for screening upregulated and downregulated lncRNAs or mRNAs.

### RNA extraction, RT-qPCR, western blotting, and transwell assay

RNA extraction, RT-qPCR, western blotting, and transwell assay were performed as described previously [18]. The following antibodies were used for western blot analysis: GAPDH (CST), p65 (CST), p-p65 (CST), IκBα (CST), p-IκBα (CST), MMP14 (CST), histone 3 (Abcam), and tubulin (Abcam). The primers used for PCR and the sequences of the shRNAs used in the study are shown in Table S1.

### Chromatin immunoprecipitation (ChIP) assay

KYSE30 cells treated with PBS or recombinant TGF-β1 for 30 min were used to perform ChIP assay using an anti-Smad2/3 antibody (CST) and an EZ-Magna ChIP Chromatin Immunoprecipitation Kit (Millipore), according to the manufacturer’s instructions. The chromatin fraction isolated by ChIP was analyzed by qRT-PCR with primers specific for the promoter region of NKILA. The Smad2/3 binding motifs were predicted by JASPAR (http://jaspar.genereg.net/). The ChIP-qPCR primer is shown in Table S1.

### Lung colonization assay

Female SCID-beige mice (aged 4–5 weeks) were used for the animal studies. Forty mice were divided into four groups (10 mice per group) and administered tail-vein injections of 1 × 10^6^ KYSE30 cells containing different plasmids (vector, NKILA, shvec, and sh1). Seven weeks later, the mice were sacrificed using CO_2_ anesthesia, and the numbers of tumor nodules on their lungs were counted after the excised tissues were soaked with picric acid and embedded in paraffin for hematoxylin and eosin (H&E) staining.

### Statistical analysis

Statistical analyses were performed using SPSS version 20.0 (SPSS, Chicago, IL, USA). Chi-square tests were used to analyze the relationships between NKILA expression levels and patient clinicopathological characteristics. The differences in NKILA expression between the experimental groups were evaluated by the Mann–Whitney *U* test, and the difference in NKILA expression between the tumor and paired nontumor tissues was evaluated by the Wilcoxon matched-pairs signed-rank test. The results of the cell experiments were presented as the mean and SD from three independent experiments, and the differences among the groups were analyzed by an independent samples Student’s *t* test. Differences were considered significant at *p* < 0.05.

## Results

### LncRNA NKILA is upregulated by TGF-β

**Fig. 1:**
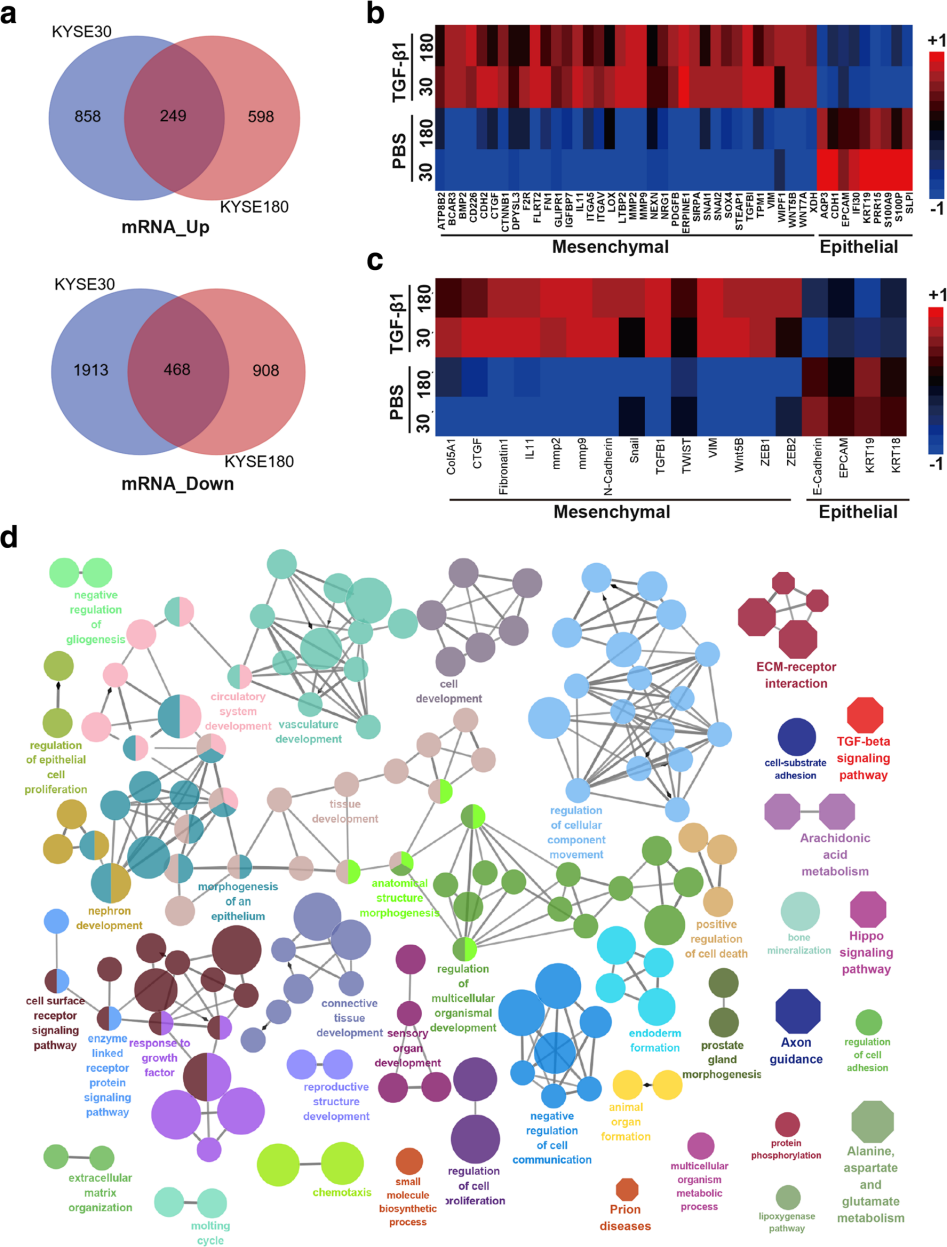
TGF-β-induced EMT markers in KYSE30 and KYSE180 cells. **a** Venn diagram of the mRNAs that are differentially expressed between KYSE30 and KYSE180 cells treated with or without TGF-β, as demonstrated by RNA-seq. **b** Heat map representation of RNA-seq data pertaining to the expression levels of EMT-related genes in KYSE30 and KYSE180 cells treated with TGF-β1 for 72 h and control cells. **c** Heat map of the expression of EMT-related genes in KYSE30 and KYSE180 cells treated with TGF-β1 for 72 h and control cells, as demonstrated by RT-qPCR. **d** Functionally grouped network in which enriched KEGG pathways and GO biological function terms served as nodes linked based on the kappa score levels (≥ 0.55). Only the label of the most significant term in each group is shown. The KEGG pathway and GO biological functional terms were enriched by the Cytoscape Cluego plugin. Octagon represents the KEGG pathway. Ellipse represents the GO terms. The node size represents the significance of the term enrichment; thus, the larger the node size, the smaller the corrected *p* value. Different colors represent different functional groups

To identify the lncRNAs that are regulated by TGF-β and mediate the regulatory effects of TGF-β on cell metastasis, we treated KYSE30 and KYSE180 ESCC cells with TGF-β1 continuously for 72 h. The treatment caused the cells to undergo EMT, based on our subsequent observation that the cells had a spindle-shaped appearance (Fig. S1A), and promoted cell migration and invasion, as demonstrated by the wound-healing (Fig. S1B) and transwell (Fig. S1C and D) assays, respectively. We then used whole-transcriptome RNA-seq to compare mRNA and lncRNA expression levels between the TGF-β1-treated and untreated cell lines. We found that 249 mRNAs were upregulated, and 468 mRNAs were downregulated in TGF-β1-treated KYSE30 and KYSE180 cells compared with the corresponding untreated cells (Fig. 1a). The RNA-seq results indicated that TGF-β1 treatment led to EMT gene expression signature with high expression level of mesenchymal markers and low expression level of epithelial markers (Fig. 1b). To evaluate RNA-seq accuracy, we confirmed the expression of several essential EMT markers, including CDH2, VIM, ZEB1, SNAI1, and CDH1, by RT-qPCR (Fig. 1c). Functionally grouped networks [19] of enriched KEGG pathways and GO biological functional terms indicated that many TGF-β-responsive signaling pathways and biological processes were significantly enriched in the TGF-β1-treated cell lines (Fig. 1d and Fig. S2), confirming that the RNA-Seq results were reliable.

**Fig. 2:**
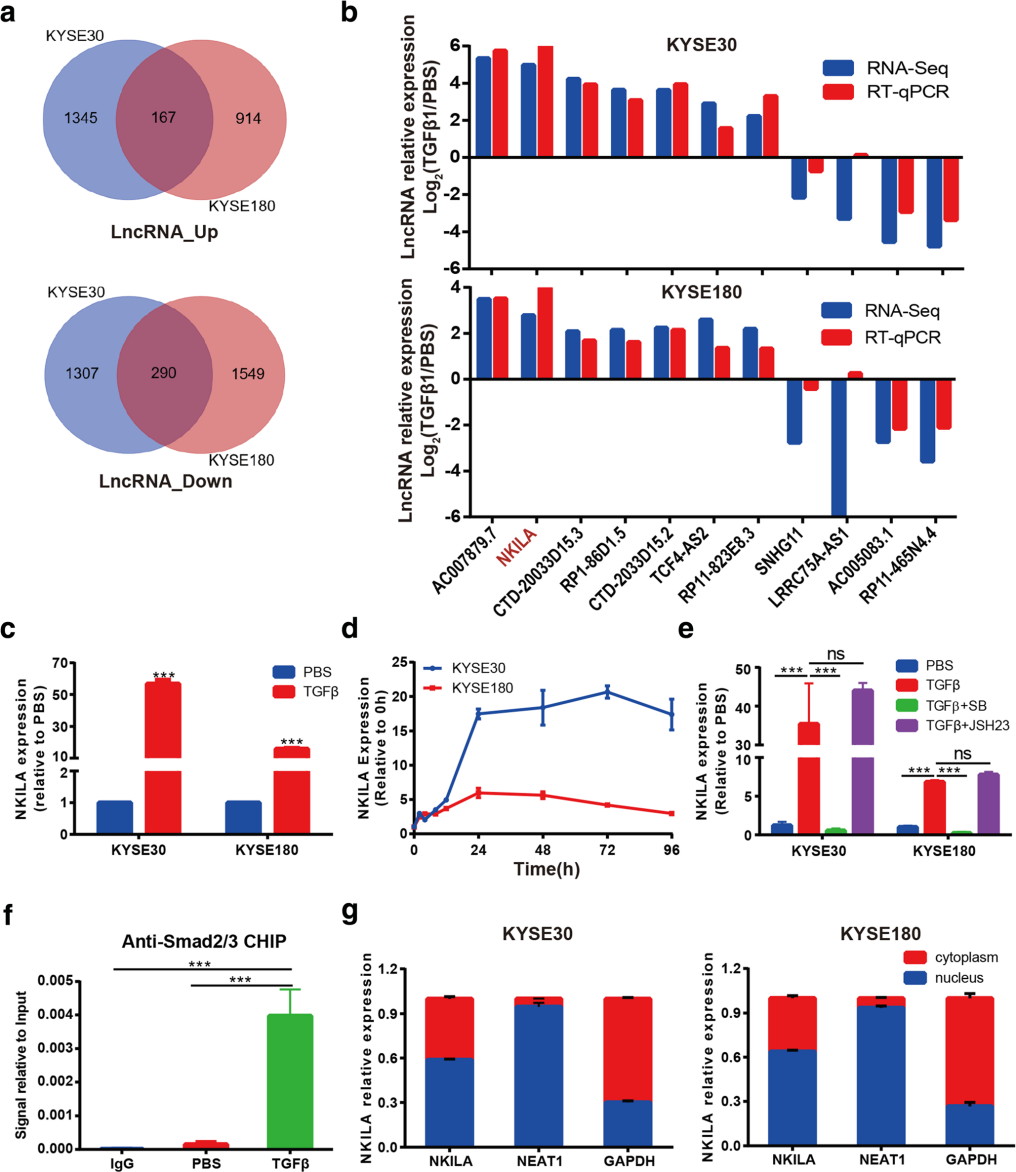
NKILA is upregulated by the classic TGF-β pathway. **a** Venn diagram of the lncRNAs that are differentially expressed between KYSE30 and KYSE180 cells treated with or without TGF-β, as demonstrated by RNA-seq. **b** The expression levels of the top 11 differentially expressed lncRNAs detected by RNA-seq were validated by RT-qPCR in KYSE30 and KYSE180 cells treated with TGF-β1 for 72 h and control cells. **c** Relative expression levels of NKILA in KYSE30 or KYSE180 cells treated with or without TGF-β1, as measured by qRT-PCR. **d** Kinetics of NKILA expression in KYSE30 and KYSE180 cells following TGF-β1 stimulation. **e** NKILA expression, as demonstrated by qRT-PCR, in KYSE30 and KYSE180 cells treated with TGF-β1 and with or without the TGF-β inhibitor SB505124 (SB) or the NF-κB inhibitor JSH-23 (JSH). **f** The Smad2/3 complex localizes to the NKILA promoter in KYSE30 cells treated with TGF-β1 or PBS for 30 min, as determined by the ChIP assay. **g** Subcellular localization, as assessed by RT-qPCR, indicated that NKILA was expressed in the nucleus and cytoplasm. GAPDH and NEAT1 RNA were used as fractionation indicators. Data are shown as the mean ± SD; *n* = 3. ***p < 0.001, ns means no significant difference (Student’s *t* test)

We found that 167 lncRNAs were upregulated, and 290 lncRNAs were downregulated in both TGF-β1-treated cell lines compared with the corresponding untreated cell lines (Fig. 2a). We then limited our search to lncRNAs with FPKM values ≥ 1 and expression levels differing by more than ≥ 4.0-fold between treated and untreated cells. We identified seven lncRNAs whose expression was upregulated and four lncRNAs whose expression was downregulated in treated cells compared with untreated cells and verified the expression most of the lncRNAs by RT-qPCR (Fig. 2b). Of those lncRNAs, the lncRNA whose expression was most upregulated in TGF-β1-treated cells was the lncRNA NKILA (Fig. 2b), whose expression was consistently found to be upregulated by more than 10-fold in both ESCC cell lines compared with the corresponding untreated cell lines (Fig. 2c). NKILA expression peaked at 24 h after TGF-β1 treatment and remained elevated for up to 96 h after treatment in KYSE30 and KYSE180 cells (Fig. 2d). NKILA is a lncRNA encoded by a gene on chromosome 20q13 and was initially identified as an NF-κB-induced lncRNA in breast cancer [20]. To determine whether TGF-β signaling is responsible for NKILA expression, we used the TGF-β receptor inhibitor SB505124 and the NF-κB nuclear translocation inhibitor JSH-23 to abrogate the effects of TGF-β1 treatment on NKILA expression. The results showed that SB505124 completely inhibited TGF-β1-induced NKILA expression in KYSE30 and KYSE180 cells but not JSH-23 (Fig. 2e).

To investigate whether NKILA is regulated by the classical TGF-β pathway, we performed ChIP assay using anti-Smad2/3 antibodies. We found that TGF-β1 treatment led to a significant increase of enriched NKILA promoter sequence, which implied that the Smad2/3 complex was recruited to the promoter of the NKILA gene by TGF-β1 treatment (Fig. 2f). We also observed that the Smad3 phosphorylation selective inhibitor SIS3 could restore back TGFβ-induced NKILA expression (Fig. S3). In addition, nuclear and cytosolic fraction isolation studies and RT-qPCR showed that NKILA was expressed in the nucleus and cytoplasm simultaneously in KYSE30 and KYSE180 cells, a result that was inconsistent with those of previous reports regarding NKILA expression in breast cancer and was probably due to cell-specific differences in NKILA regulation. GAPDH and nucleic RNA NEAT1 were used as fractionation indicators (Fig. 2g). Taken together, these results suggested that NKILA was dramatically upregulated by the classical TGF-β signaling pathway in ESCC cells.

### NKILA negatively regulated tumor migration and invasion in vitro

**Fig. 3:**
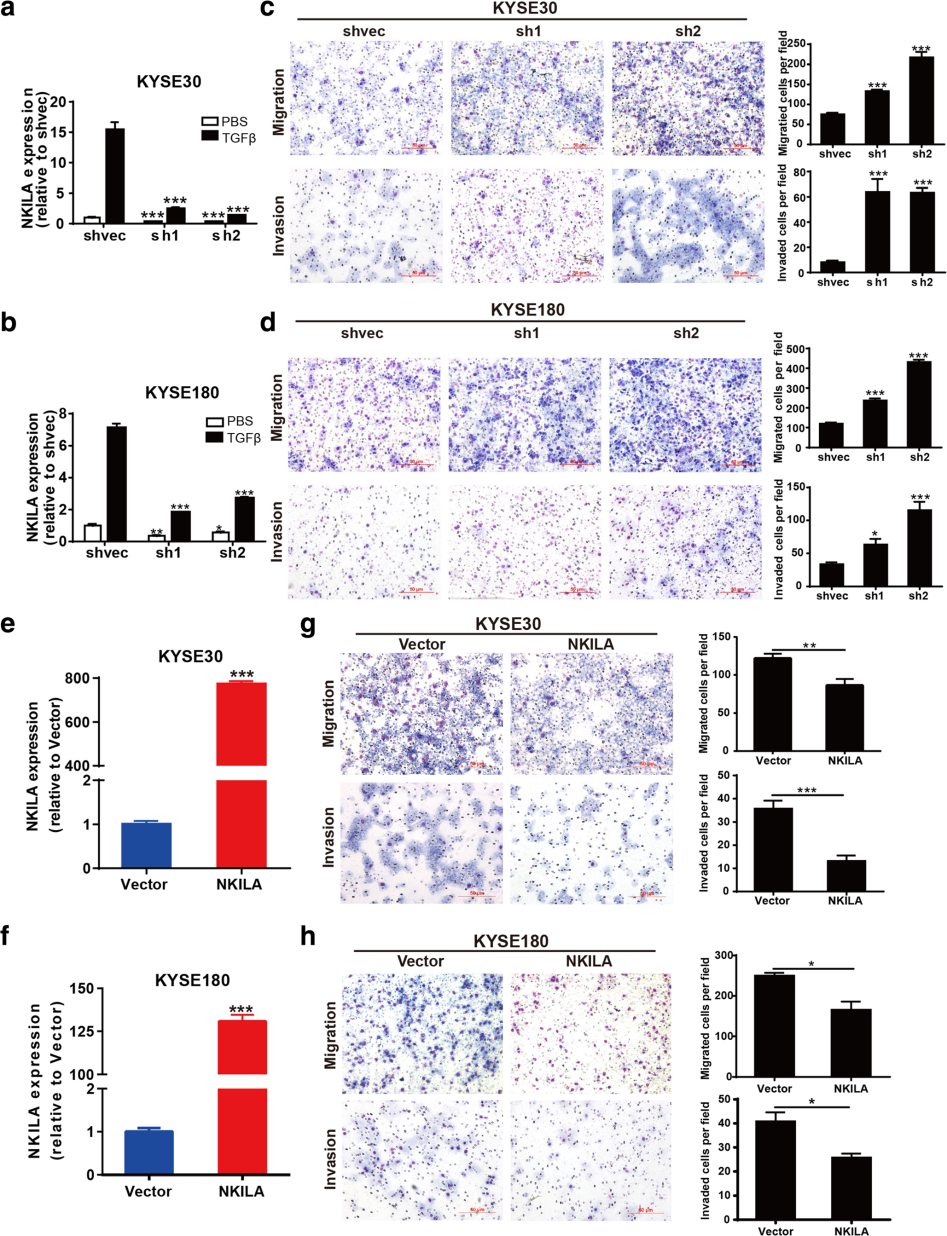
NKILA negatively regulated cell migration and invasion in vitro. **a**, **b** NKILA expression in stable knockdown cell clones. **c**, **d** The indicated KYSE30 cell clones (**c**) or KYSE180 cell clones (**d**) were added to the top of transwell apparatuses coated with or without Matrigel. The number of migrating or invading cells per field was counted after 24 h. **e**, **f** NKILA expression in stable NKILA-overexpressing or mock-vehicle control-transfected KYSE30 and KYSE180 cell clones. **g**, **h** The migration and invasion ability of NKILA-overexpressing KYSE30 (**g**) and KYSE180 (**h**) cells, as well as that of mock-vehicle control-transfected cells, was detected by transwell assay. The numbers of migrating and invading cells were compared between the groups. Data are shown as the mean ± SD, *n* = 3. *p < 0.05, **p < 0.01, ***p < 0.001 (Student’s *t* test)

Given the involvement of NKILA in the TGF-β signaling, which plays a vital role in cell migration and invasion, we speculated that NKILA plays an important role in tumor metastasis. First, we generated two NKILA-knockdown plasmids (named sh1 and sh2, respectively) and assessed the efficiency with which the plasmids knocked down NKILA expression via RT-qPCR. Both knockdown cell clones displayed decreases in NKILA expression of more than 70% regardless of whether the cells were treated with TGF-β1 (Fig. 3a, b). Then, we performed transwell assay to investigate the effects of NKILA expression on the malignant behavior of ESCC cells. As shown in Fig. 3c, d, the migration and invasion abilities of KYSE30 and KYSE180 cells were significantly enhanced when NKILA expression levels were decreased. Conversely, NKILA expression was significantly increased in KYSE30 and KYSE180 cells with stable NKILA overexpression compared with cells transfected with mock-vehicle controls (Fig. 3e, f). In addition, NKILA overexpression significantly suppressed the migration and invasion ability of both ESCC cell lines (Fig. 3g, h).

### NKILA inhibited tumor metastasis in vivo

**Fig. 4:**
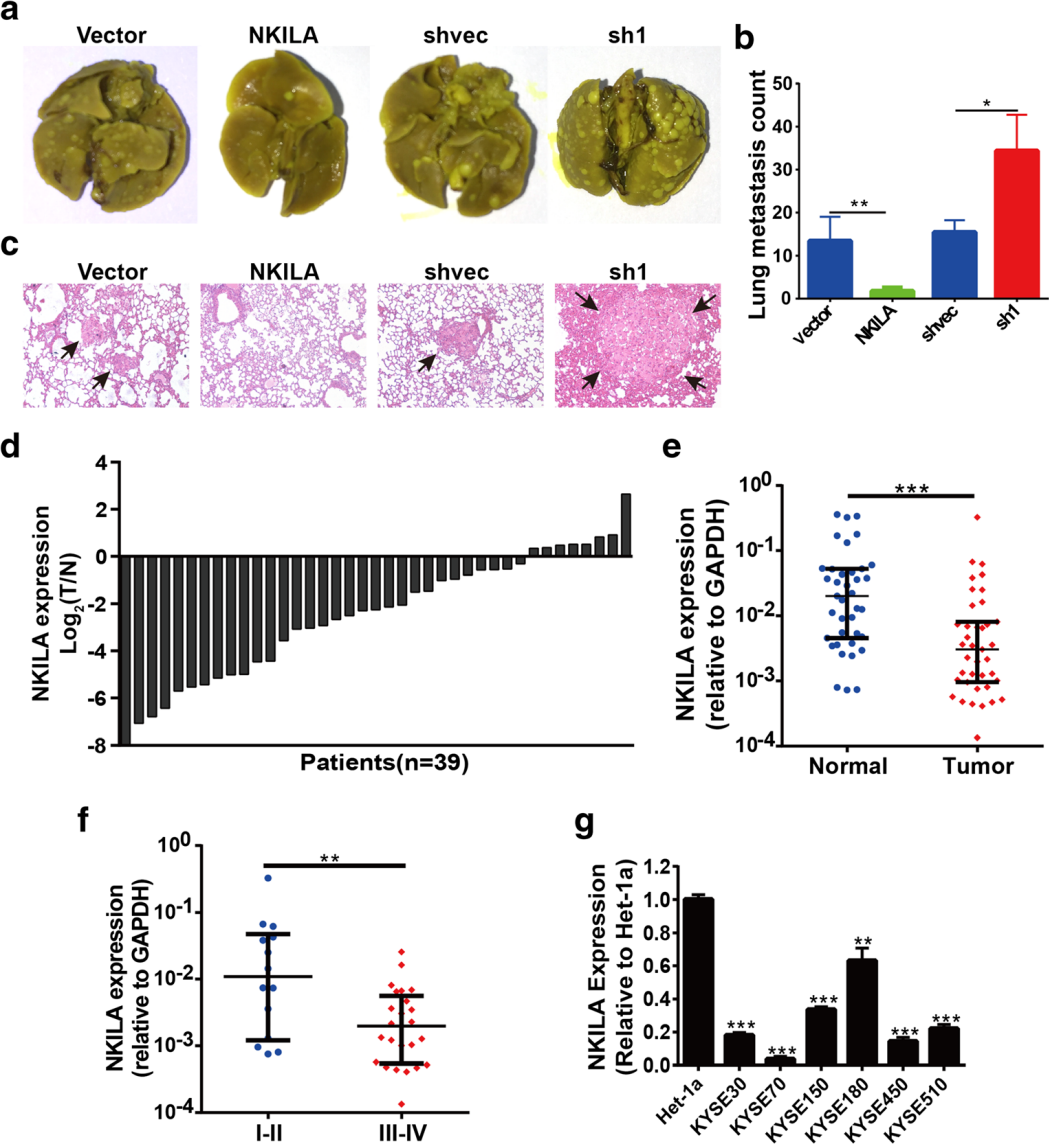
NKILA was negatively associated with malignant progression in vivo. **a** Representative images of isolated lungs from each group are shown. **b** The numbers of metastatic nodules in the lungs at **a** 7 weeks after tail vein injection were counted. **c** Representative images of H&E-stained lung sections from each group are shown. **d** The relative expression levels of NKILA in tumor tissues and adjacent normal tissues from 39 patients with ESCC are shown as log_2_(*T*/*N*) = log_2_(*T*_NKILA_/*N*_NKILA_). Each column represents a patient. **e** NKILA expression (2^−ΔCT^) in 39 tumor tissues was compared with that in paired adjacent noncancerous lung tissues. **f** NKILA expression levels were compared between 14 patients with early stage (I and II) disease and 25 patients with advanced stage (III and IV) disease. Data are shown as medians with interquartile ranges, and the Mann–Whitney *U* test was used. **g** NKILA expression levels in the esophageal epithelial cell line Het-1a and six ESCC cell lines. GAPDH was the loading control. Data are shown as the mean ± SD, *n* = 3. **p < 0.01, ***p < 0.001 (Student’s *t* test)

To evaluate the effect of NKILA expression on ESCC cell metastasis in vivo further, we performed lung colonization assay by injecting cells directly into the tail veins of non-obese diabetic (NOD)-SCID mice. The number of metastatic nodules in the lungs was counted 7 weeks after tail vein injection. NKILA-overexpressing cells displayed decreased lung colonization rates and formed less metastatic tumors in the lung, whereas NKILA-knockdown cells displayed significantly increased metastatic nodule formation compared with mock-vehicle control-transfected cells (Fig. 4a, b). H&E-stained images of lung tissues isolated from the mice are shown in Fig. 4c. These data demonstrated that NKILA restrained tumor metastasis in vivo.

### NKILA was downregulated in the tumor tissues of patients with ESCC

NKILA expression levels in tumor tissues and matched adjacent normal tissues from 39 patients with ESCC were measured by RT-qPCR, which showed that NKILA expression was significantly reduced in ESCC tissues compared with adjacent normal esophageal tissues (*p* < 0.001, Fig. 4d, e). In addition, NKILA expression levels were significantly decreased in advanced tumor tissues compared with early stage tumor tissues (*p* < 0.01, Fig. 4f). NKILA expression was also lower in tumor tissues with lymph node metastasis than in tumor tissues without lymph node metastasis, but the difference between the two groups was not significant (*p* = 0.076, Fig. S4), perhaps because the sizes of the samples were small. The 39 patients with ESCC were classified into the following two groups according to the NKILA expression level in their tumor tissues: a higher NKILA expression group (*n* = 19, NKILA expression level > median expression level) and a low NKILA expression group (*n* = 17, NKILA expression level ≤ median level). NKILA downregulation in ESCC was significantly associated with *T* stage (*p* < 0.05) and clinical stage (p < 0.05). However, there were no correlations between NKILA expression and age, gender, smoking, pathologic grade, and tumor size (*p* > 0.05) (Table S2). In addition, NKILA expression was significantly downregulated in the abovementioned six ESCC cell lines compared with the human esophageal epithelial cell line Het-1a (Fig. 4g). All these data suggested that NKILA expression was significantly downregulated in ESCC tumor tissues and implicated NKLIA in ESCC tumorigenesis and progression.

### NKILA inhibited IκBα phosphorylation and NF-κB activation in ESCC cells

**Fig. 5:**
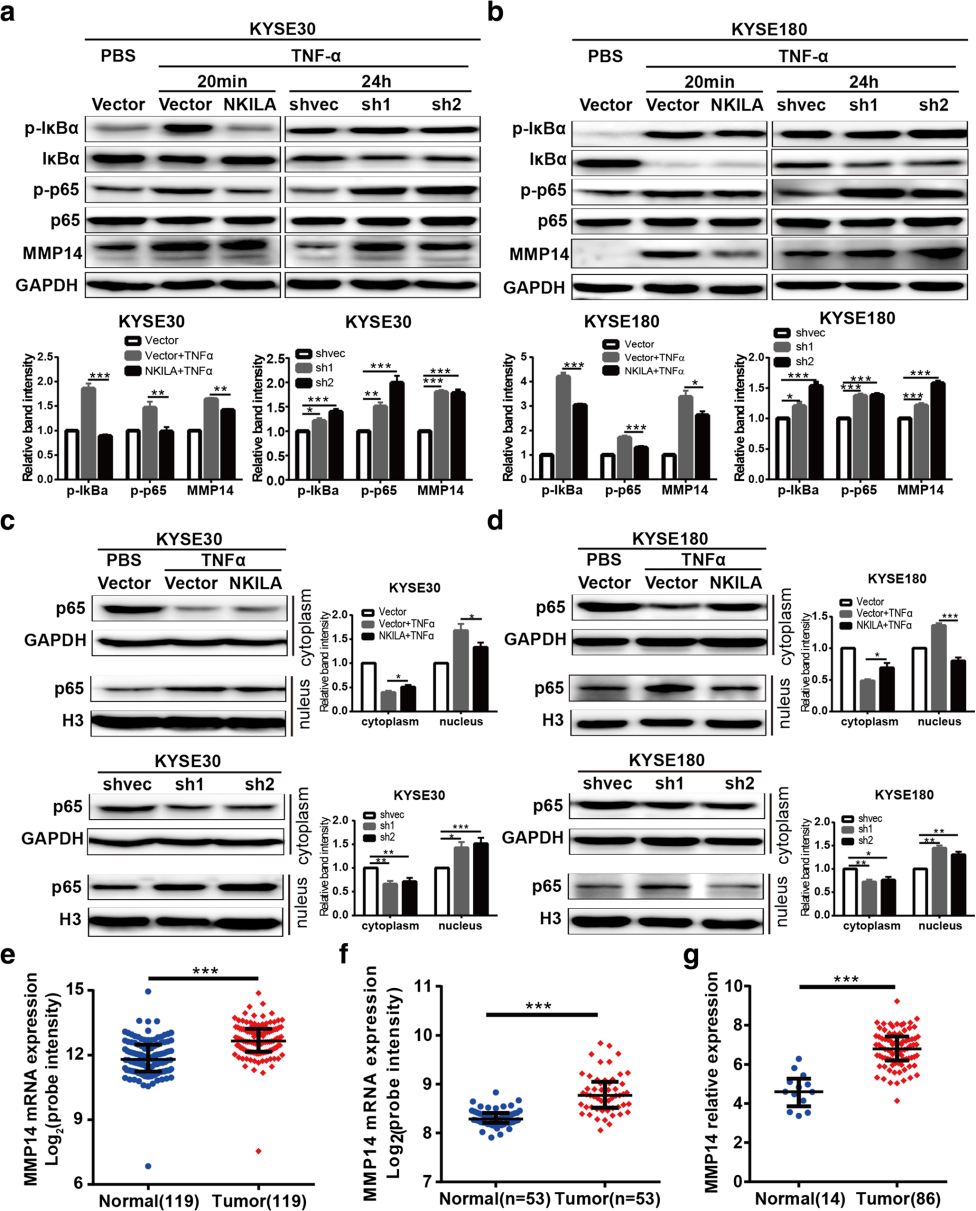
NKILA suppressed NF-κB signaling activation and MMP14 expression. **a**, **b** Western blotting showing the expression of crucial components (IκB, p-IκB, p65, p-p65) of the NF-κB signaling pathway and MMP14 in stable KYSE30 (**a**) or KYSE180 (**b**) cell clones treated with or without TNF-α for the indicated time. The left panel features representative images, and the right panel features a statistical diagram. GAPDH was the loading control. **c**, **d** Western blotting for nuclear and cytoplasmic p65 in stable KYSE30 (**c**) and KYSE180 (**d**) cell clones treated with or without TNF-α. GAPDH and histone 3 (H3) were the loading controls for the cytoplasm and nucleus, respectively. The left panel features representative images, and the right panel features a statistical diagram. Data are shown as the mean ± SD, *n* = 3. **e**–**g** MMP14 mRNA expression levels were calculated from three data sets, GSE53625, GSE23400, and TCGA ESCC RNA-seq data (http://xena.ucsc.edu/). The former two data sets included cDNA microarray data for matched normal and ESCC tissues. *p < 0.05, **p < 0.01, ***p < 0.001 (Student’s *t* test)

NKILA has been reported to interact with and influence the activation of NF-κB signaling in breast cancer [20]. We detected IκBα and p65 phosphorylation levels in NKILA-overexpressing and silenced ESCC cells and found that TNF-α-induced IκBα and p65 phosphorylation was inhibited by ectopic NKILA expression in KYSE30 and KYSE180 cells but was enhanced by NKLIA silencing in both cell lines (Fig. 5a, b). Furthermore, we found that ectopic NKILA expression markedly inhibited TNF-α-induced p65 nuclear translocation in KYSE30 and KYSE180 cells compared with mock-vehicle control-transfected cells, while NKILA silencing significantly prolonged p65 translocation (to 24 h) in KYSE30 and KYSE180 cells compared with mock-vehicle control-transfected cells, results consistent with those mentioned above (Fig. 5c, d). These results suggested that NKILA suppressed NF-κB activation by inhibiting IKK-induced IκBα phosphorylation.

### NKILA inhibited ESCC cell migration and invasion through NF-κB/MMP14 signaling

**Fig. 6:**
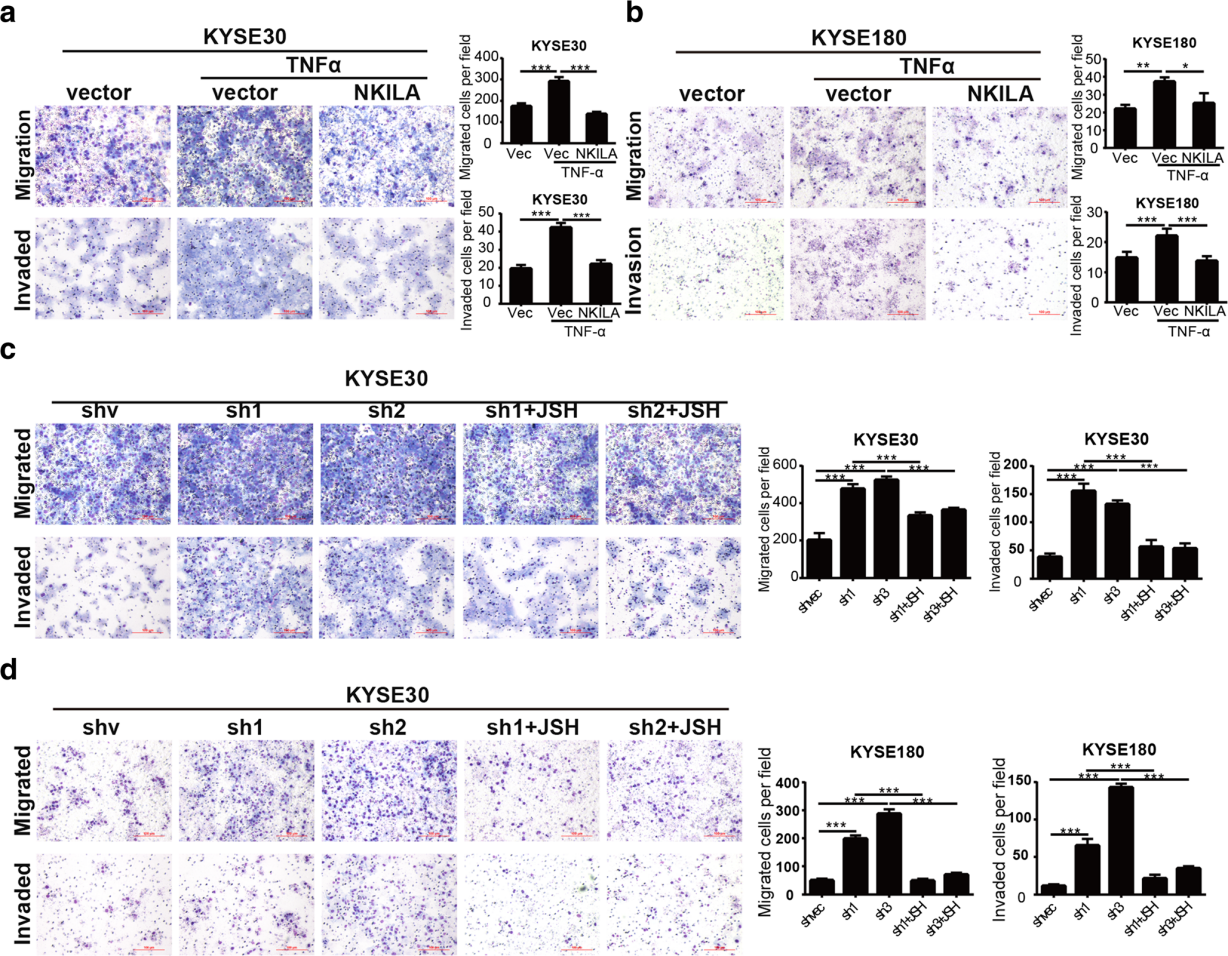
NKILA regulates ESCC cell migration and invasion via the NF-κB/MMP14 pathway. **a**, **b** Migration and invasion of KYSE30 and KYSE180 cells stably expressing NKILA or mock-vehicle controls after treatment with or without TNF-α, as measured by Chamber assay. **e**, **f** Migration and invasion of stable KYSE30 and KYSE180 NKILA-knockdown or mock-vehicle control cell clones exposed to TNF-α and treated with or without JSH-23 (JSH), as measured by Chamber assay. The left panel features representative images, and the right panel features a statistical diagram. Data are shown as the mean ± SD, *n* = 3. *p < 0.05, **p < 0.01, ***p < 0.001 (Student’s *t* test)

Matrix metalloproteinases (MMPs), which play a central role in extracellular matrix turnover and cancer cell migration and invasion [21], are one of the most important downstream regulators of NF-κB-induced cell metastasis. To determine how NKILA regulates cell migration and invasion, we detected MMP2, MMP9, and MMP14 protein expression levels in NKILA-overexpressing or silenced ESCC cells. We found that MMP14 expression levels were remarkably attenuated by ectopic NKILA expression. In contrast, silencing NKILA elevated MMP14 expression levels in KYSE30 and KYSE180 cells (Fig. 5a, b). Furthermore, NKILA-induced changes in MMP14 expression levels were completely abrogated by the NF-κB translocation inhibitor JSH-23 (Fig. S5). Coincidentally, our mRNA microarray data [22] pertaining to 119 pared tissues from patients with ESCC (Fig. 5e), microarray data collected by Hua Su [23] (Fig. 5f), and ESCC RNA-seq data from TCGA (Fig. 5g) showed that MMP14 expression was significantly upregulated in ESCC tissues compared with paired normal tissues. To determine whether NF-κB/MMP14 signaling is responsible for NKILA-regulated cell migration and invasion, we used TNF-α to induce NF-κB pathway activation and consequently promote KYSE30 and KYSE180 cell migration and invasion. The results showed that the decreases in migration and invasion ability caused by NKILA overexpression were reversed by TNF-α stimulation (Fig. 6a, b). Conversely, the NF-κB translocation inhibitor JSH-23 abrogated the enhancements of cell migration and invasion ability induced by NKILA silencing (Fig. 6c, d). Collectively, all these results suggested that NKILA regulated ESCC cell migration and invasion through NF-κB/MMP14 signaling.

## Discussion

Numerous studies have found that the TGF-β signaling pathway plays vital roles in regulating extracellular matrix (ECM) production and tumor metastasis; however, the mechanisms underlying its effects remain unclear. In recent years, an increasing number of lncRNAs have been found to be involved in the TGF-β signaling pathway in various cancers [24] and to complement the complicated biological mechanisms through which TGF-β signaling regulates cancer development and progression. TGF-β signaling is a key player in the genesis and development of ESCC, and the output of the TGFβ response is highly contextual. Given the growing number of lncRNAs reported to be involved in ESCC [25–29], we speculated that lncRNA may also be involved in the TGF-β signaling pathway. In our study, we found that the lncRNA NKILA was dramatically induced by TGF-β1 in KYSE30 and KYSE180 ESCC cells via RNA-seq, findings that were validated by RT-qPCR. NKILA was reported to be upregulated by more than 12-fold by TNF-α- and IL-1β-induced NF-κB activation in breast cancer [20]. However, in this study, NKILA expression levels only doubled in ESCC cells treated with TNF-α or IL-1β. More importantly, TGF-β1-induced NKILA expression could not be reversed by the NF-κB nuclear translocation inhibitor JSH-23, while TNF-α- or IL1β-induced NKILA upregulation was completely inhibited by the TGF-β receptor inhibitor SB505124 (Fig. S6). Based on these results, as well as the ChIP assay results, which are shown in Fig. 2f, we concluded that NKILA is directly upregulated by the TGF-β classical signaling pathway, which suggested that NKILA may be involved in TGF-β signaling in ESCC cells. We performed loss- and gain-of-function assays to investigate the role of NKILA in tumor cell malignant behavior. The results showed that NKILA attenuated ESCC cell migration and invasion in vitro and lung colonization in vivo. Moreover, NKILA expression levels, which were remarkably reduced in ESCC tumor tissues compared with adjacent noncancerous tissues, were negatively correlated with TNM stages and lymph node metastasis (no statistical significance). These findings suggested that NKILA was involved in TGF-β signaling and acts as a tumor suppressor in ESCC.

Metastasis is the leading cause of death in patients with cancer. Invasive cell migration through tissue barriers requires pericellular remodeling of the ECM, a process executed by cell-surface proteases, particularly MMPs. Several ECM-degrading proteolytic enzymes, such as MMP-1, 2, 13, and 14 and cathepsins B, K, and L, have been implicated in this process; however, MMP-14 may be the critical and rate-limiting enzyme in collagen turnover [21, 30, 31]. Previous studies and our microarray data showed that MMP14 expression is upregulated in ESCC tumor tissues and is associated with a poor prognosis [32–36], findings that are attributable at least in part to the metastasis-promotion function of MMP14 in ESCC cells. In the present study, we found that NKILA decreased MMP14 expression levels by mediating IκBα phosphorylation and NF-κB translocation to the nucleus and consequently weakened ESCC cell migration and invasion ability. In addition, the effects of NKILA on MMP14 expression and ESCC cell metastatic potential were abrogated by the NF-κB translocation inhibitor JSH-23 in KYSE30 and KYSE180 cells. Collectively, these findings indicate that NKILA-regulated migration and invasion is mediated by the NF-κB/MMP14 signaling pathway in ESCC cells.

TGF-β can be secreted by tumor-associated macrophages (TAMs) and cancer-associated fibroblasts (CAFS), as well as cancer cells, and is constitutively active in many tumors [37]. Mounting numbers of studies have established that the TGF-β pathway is an essential factor in cancer development and plays either a tumor-suppressing or a tumor-promoting role depending on the concentrations of different ligands. TGF-β signaling pathway activity duration and intensity are critical determinants of the biological responses of TGF-β family members [38, 39]. Several negative regulators of the TGF-β pathway, for example, prostate transmembrane protein androgen-induced 1 (PMEPA1) [40] and thrombospondin-1 (THBS1) [41, 42], certain I-Smads (i.e., Smad6 and Smad7), Smad ubiquitination regulatory factors (Smurfs) [43], and TG-interacting factor (TGIF) [44], have been shown to be tumor suppressors. Many of these regulators are transcribed by TGF-β but participate in negative feedback loops to prevent sustained or excessive TGF-β pathway activation. We found that NKILA, a TGF-β-induced lncRNA, is also involved in these negative-feedback networks and serves as tumor suppressor by inhibiting ESCC progression and metastasis. In the present study, we found that NKILA expression was decreased in advanced ESCC tumor tissues and that NKILA inhibited ESCC cell metastasis in vitro and in vivo and thus compliments the complicated networks through which TGF-β signaling regulates cancer progression and metastasis.

In summary, our research demonstrated that NKILA acts downstream of the TGF-β signaling pathway and inhibits cell invasion and metastasis by inhibiting IκBα phosphorylation and NF-κB activation and, consequently, suppressing MMP14 expression. Moreover, NKILA expression levels are decreased in the advanced tumor tissues of patients with ESCC. More samples are needed to evaluate the role of NKILA in ESCC to determine its clinical value. The observations discussed herein indicate that NKILA has potential as a target in the treatment of ESCC.

## Electronic supplementary material

ESM 1 (DOCX 2535 kb)
