# Supplementary material for: TGF-β-induced NKILA inhibits ESCC cell migration and invasion through NF-κB/MMP14 signaling
Source: J Mol Med (Berl). 2018 Jan 29;96(3):301–13. doi: 10.1007/s00109-018-1621-1 (PMC5859688; doi:10.1007/s00109-018-1621-1)
Supplement: Supplementary file 1 — (DOCX 2535 kb) [file 109_2018_1621_MOESM1_ESM.docx]

**Supplementary Files**

| **Name** | **Direction** | **sequence (5'-3')** |
| --- | --- | --- |
| GAPDH | Forward | CCTGGTATGACAACGAATTTG |
|  | Reverse | CAGTGAGGGTCTCTCTCTTCC |
| Col5A1 | Forward | CACAACTTGCCTGATGGAATAACA |
|  | Reverse | GCAGGGTACAGCTGCTTGGT |
| CTGF | Forward | CACCCGGGTTACCAATGACA |
|  | Reverse | TGCAGCCAGAAAGCTCAAAC |
| Fibronatin1 | Forward | CCATCGCAAACCGCTGCCAT |
|  | Reverse | AACACTTCTCAGCTATGGGCTT |
| IL11 | Forward | GCTGCAAGGTCAAGATGGTT |
|  | Reverse | GCTGGGTGGCGTTCTATC |
| mmp2 | Forward | CCGTCGCCCATCATCAAGTT |
|  | Reverse | CTGTCTGGGGCAGTCCAAAG |
| mmp9 | Forward | TGGCAGAGATGCGTGGAGA |
|  | Reverse | GGCAAGTCTTCCGAGTAGTTTT |
| N-Cadherin | Forward | TGGACCATCACTCGGCTTA |
|  | Reverse | ACACTGGCAAACCTTCACG |
| Snail | Forward | TGCGTCTGCGGAACCTG |
|  | Reverse | GGACTCTTGGTGCTTGTGGA |
| TGFB1 | Forward | CTAATGGTGGAAACCCACAACG |
|  | Reverse | TATCGCCAGGAATTGTTGCTG |
| TWIST | Forward | GTCCGCAGTCTTACGAGGAG |
|  | Reverse | TGGAGGACCTGGTAGAGGAA |
| VIM | Forward | CCTGAACCTGAGGGAAACTAA |
|  | Reverse | GCAGAAAGGCACTTGAAAGC |
| Wnt5B | Forward | ACTGCAGCACTGTGGACAAC |
|  | Reverse | GCGGTAGCCATACTCCACAT |
| ZEB1 | Forward | ACTCTGATTCTACACCGC |
|  | Reverse | TGTCACATTGATAGGGCTT |
| ZEB2 | Forward | TGAGGATGACGGTATTGC |
|  | Reverse | ATCTCGTTGTTGTGCCAG |
| E-Cadherin | Forward | GCCCCATCAGGCCTCCGTTT |
|  | Reverse | ACCTTGCCTTCTTTGTCTTTGTTGGA |
| EPCAM | Forward | GAACACTGCTGGGGTCAGAA |
|  | Reverse | TCCTTCTGAAGTGCAGTCCG |
| KRT18 | Forward | CCCTGGACGTGGAGATTG |
|  | Reverse | GCAGGAGCCTTTCACTTGG |

**Table S1 Sequences of primers and shRNAs used in this study**

**Table S1 Sequences of primers and shRNAs used in this study (continuous)**

| **Name** | **Direction** | **sequence (5'-3')** |
| --- | --- | --- |
| KRT19 | Forward | GAGGAGCTGAACAGGGAGGT |
|  | Reverse | CTGGGCTTCGATACCACTGA |
| NEAT1 | Forward | GATGCGCGCCTGGGTGTAGTT |
|  | Reverse | CATGCAGCCTGCCCCACTGT |
| AC007879.7 | Forward | AAGAGGGAGTGGGAGTTAGGC |
|  | Reverse | CTGTGGTGGGATCTGGAAGAA |
| **NKILA** | Forward | AACCAAACCTACCCACAACG |
|  | Reverse | ACCACTAAGTCAATCCCAGGTG |
| CTD-2033D15.3 | Forward | AGATGAACCACAGGCAACAA |
|  | Reverse | ACCCTCTTCCCCAGCATT |
| RP1-86D1.5 | Forward | ACTGATCACAGGTCACACAGAA |
|  | Reverse | CTTGAGGAAACCAAGTGGGAGA |
| CTD-2033D15.2 | Forward | GGGGTGAGAGGAACAACAAA |
|  | Reverse | TGTCAGGGGAGCCATACAC |
| TCF4-AS2 | Forward | TCAGGTTCTCTCTTCGTCTGC |
|  | Reverse | TTTTGTTGCTCCCACTTTCA |
| RP11-823E8.3 | Forward | GAGCGGAGCAGAGGTATT |
|  | Reverse | AGCGACTGGAACGAGAAT |
| SNHG11 | Forward | TCGAATAATGTTGTGCTGAGAC |
|  | Reverse | GACCAAGAGTGACGAGTGATG |
| LRRC75A-AS1 | Forward | TTCATTCCCGTTGTTATGGAG |
|  | Reverse | TACCCCGTCTTCAGCACTCT |
| AC005083.1 | Forward | ATCCTGCTGACTCATACAAT |
|  | Reverse | TCCTTGCGTGTTATCACAA |
| RP11-465N4.4 | Forward | GACTCCATCCAAGCATCAGG |
|  | Reverse | TTACCAGGCTCTTTCGCATT |
| ChIP-primer | Forward | CTCGGTGACACGCACTCTT |
|  | Reverse | GACACTGTCTGGTCTGCGAG |
| shRNA1 |  | GGAGAAGTCACACGTTGATTG |
| shRNA2 |  | GGCAGTAGGAAAGGAGAATTG |

**Table S2 Correlation between the clinicopathologic features and expression of NKILA**

| **Characteristics** | **NKILA relative expression N (%)** | | ***p*** |
| --- | --- | --- | --- |
|  | **Low expression (20)** | **High expression (19)** |  |
| **Age (y)** |  |  |  |
| <60 | 9(45) | 11(55) | 0.527 |
| ≥60 | 11(57.9) | 8(42.1) |  |
| **Gender** |  |  |  |
| Male | 17(54.8) | 14(45.2) | 0.451 |
| Female | 3(37.5) | 5(62.5) |  |
| **Smoking** |  |  |  |
| Smoker | 15(60) | 10(40) | 0.191 |
| Non-smoker | 5(35.7) | 9(64.3) |  |
| **Pathologic grades** |  |  |  |
| Well | 3(33.3) | 6(66.7) | 0.412 |
| Media | 12(66.7) | 8(33.3) |  |
| poor | 5(50) | 5(50) |  |
| **Tumor size** |  |  |  |
| < 6cm | 9(42.9) | 12(57.1) | 0.341 |
| ≥6cm | 11(61.1) | 7(38.9) |  |
| **T stage** |  |  |  |
| 1-2 | 0(0) | 4(100) | **0.047** |
| 3-4 | 20(57.1) | 15(42.9) |  |
| **TNM stage** |  |  |  |
| Ⅰ-Ⅱ | 4(28.6) | 10(71.4) | **0.048** |
| Ⅲ-Ⅳ | 16(64.0) | 9(36) |  |
| **Lymph node metastasis** |  |  |  |
| Negative | 5(33.3) | 10(66.7) | 0.105 |
| Positive | 15(51.3) | 9(48.7) |  |


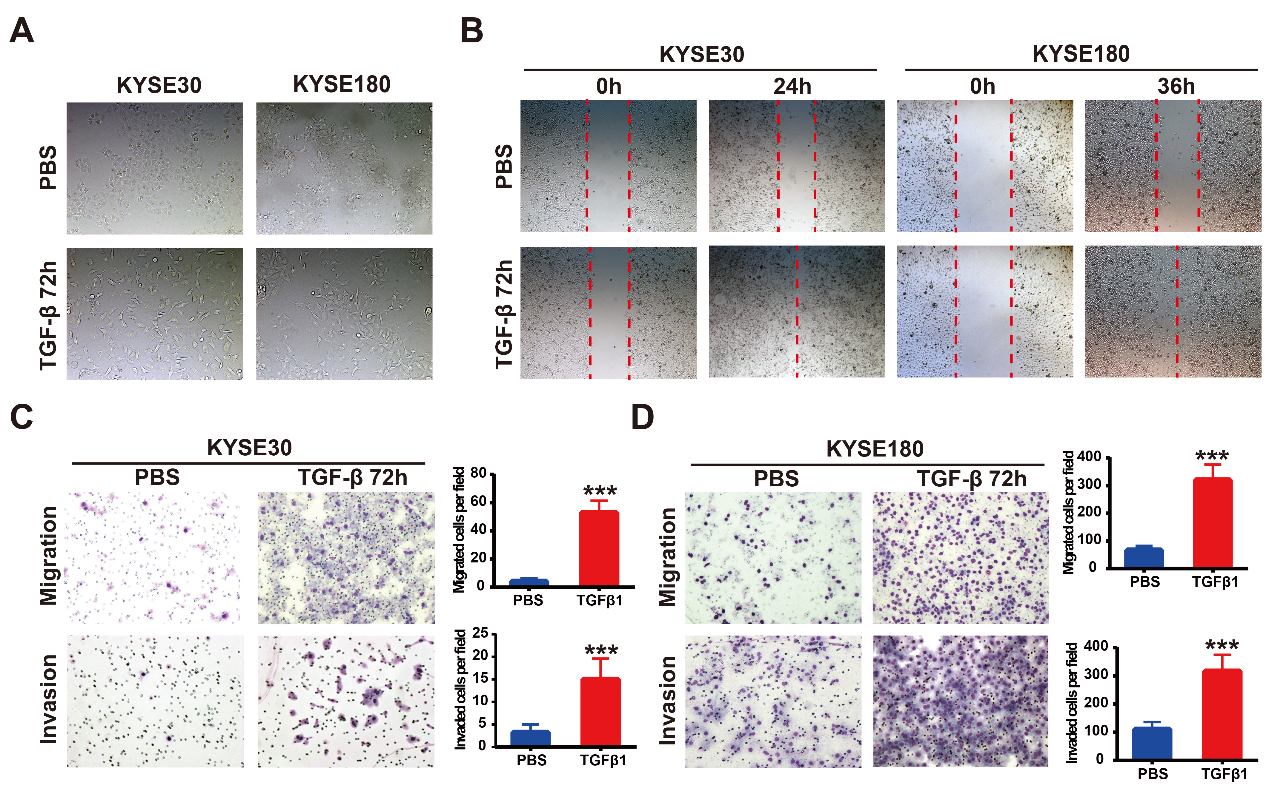
**Fig. S1. TGF-β1 treatment promoted KYSE30 and KYSE180 cell migration and invasion.** (A) Morphological changes in KYSE30 and KYSE180 cells treated with TGF-β1 for 72 h. (B) Would healing assays with KYSE30 and KYSE180 cells treated with or without 5 ng/ml TGF-β1. Cells were plated in 6-well plates, and the cell monolayer was scratched 24 h later using a 10-µl pipette tip. Wound closure was then monitored every 12 h after scratching. (C and D) Transwell assays were used to compare the migration and invasion of KYSE30 and KYSE180 cells with or without TGF-β1 treatment. The cells that migrated through the membrane or invaded the lower surface of the membrane were fixed and stained with Giemsa. Data are presented as the mean ± SD, n = 3. Student’s t-test was used to analyze the results; ***p < 0.001.


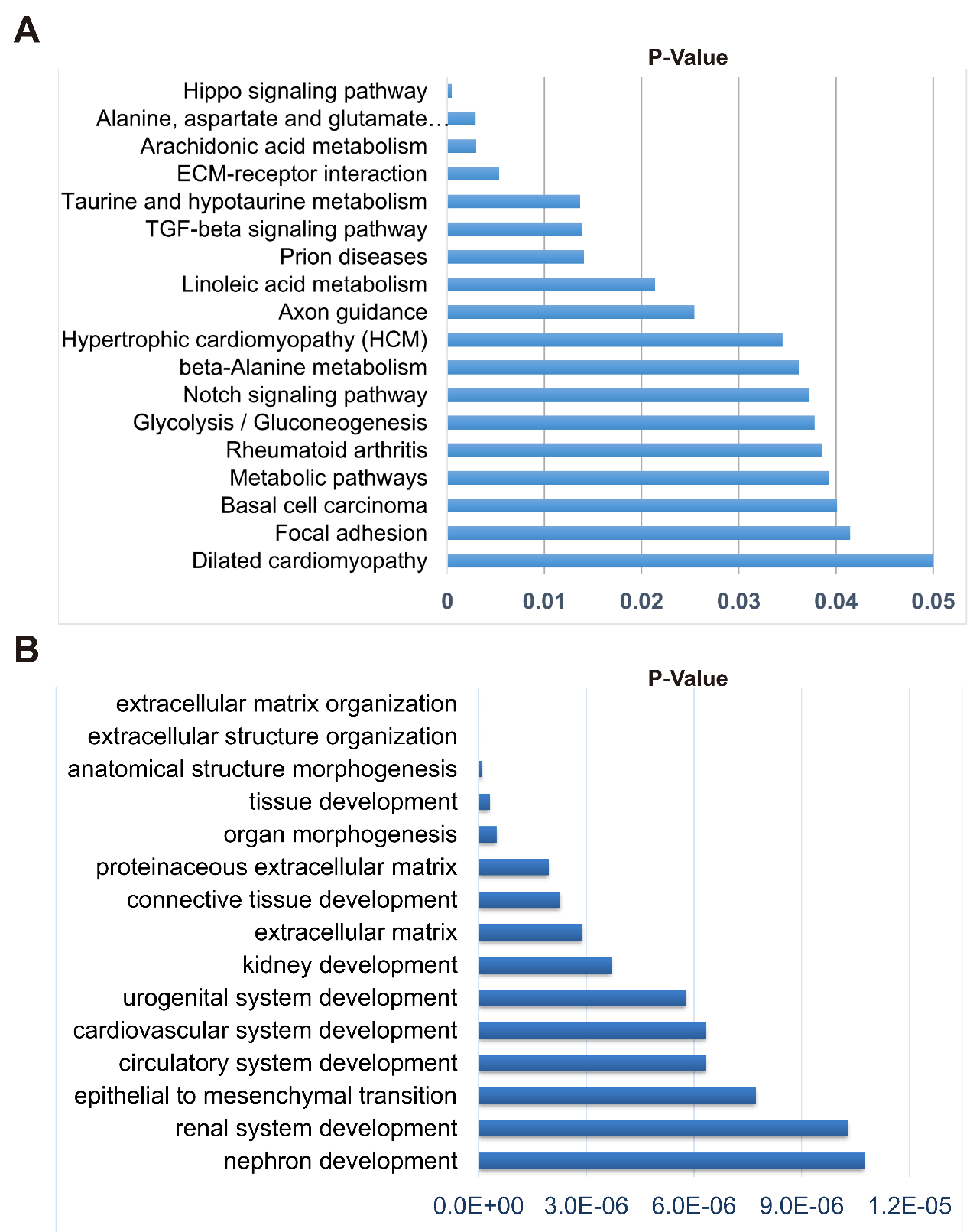


**Fig. S2 Top enriched KEGG pathways and GO terms based on the differentially expressed genes.** Top enriched KEGG pathway (A) and GO terms (B) are presented according to p-value.


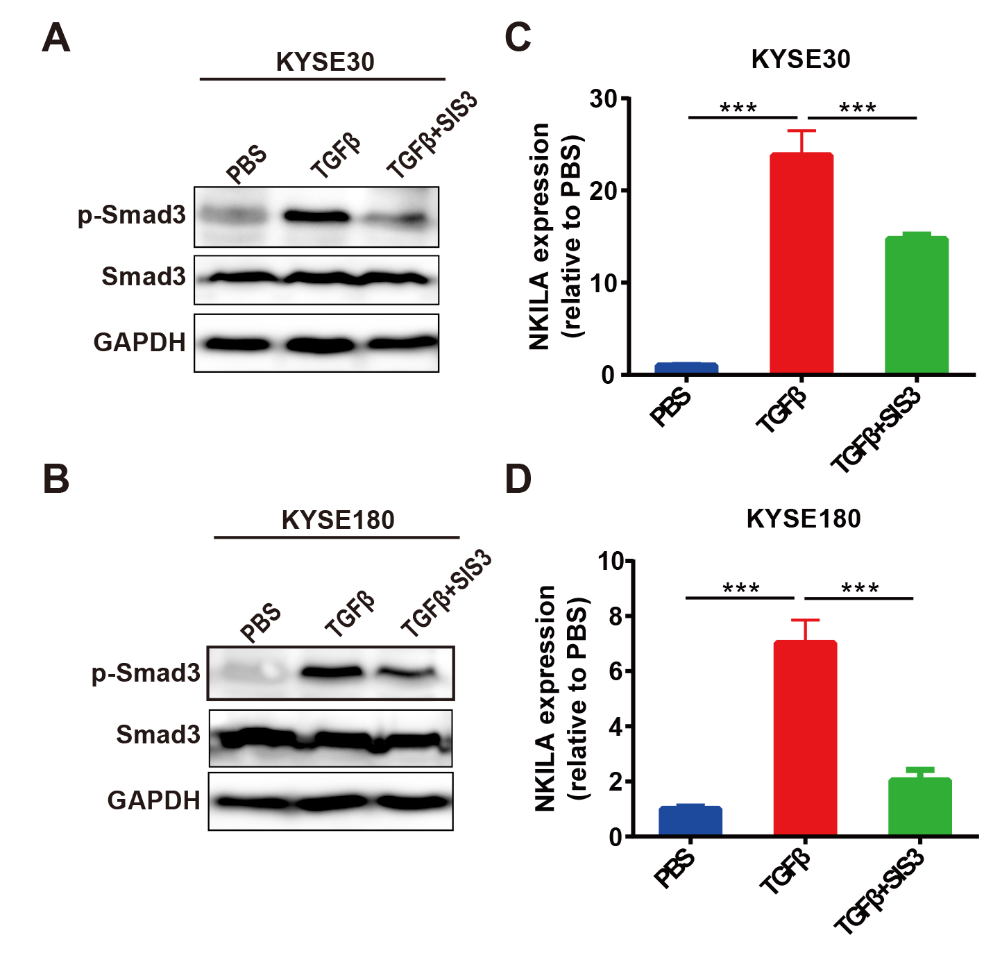
**Fig. S3 Smad3** **phosphorylation selective inhibitor SIS3 could restore TGFβ-induced NKILA expression.** TGFβ incubated KYSE30 and KYSE180 cells were treated with or without Smad3 phosphorylation selective inhibitor SIS3 (Selleck), and the expression levels of p-Smad3(A and B) and lncRNA NKILA (C and D) were measured by western blot and RT-qPCR respectively. GAPDH served as the loading control. SIS3 were added at the concentration of 10uM 30 minutes before incubating with TGFβ. Cells were collected 6 hrs later for western blot and 24 hrs later for RT-qPCR. The antibodies of p-Smad3 and total Smad3 were bought from CST. Data are presented as the mean ± SD, n = 3. Student’s t-test was used to analyze the results; ***p < 0.001.


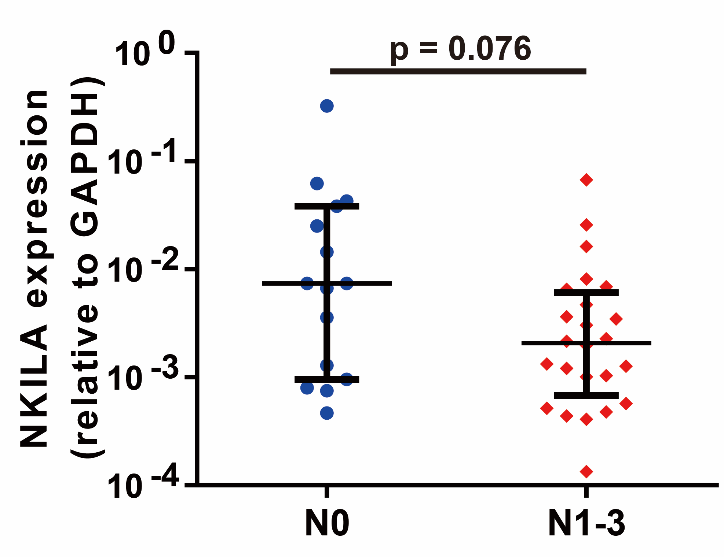
**Fig. S4 NKILA expression levels in tumor tissues with or without lymph node metastasis.** The Mann-Whitney U test was used to compare the NKILA expression level between two groups.


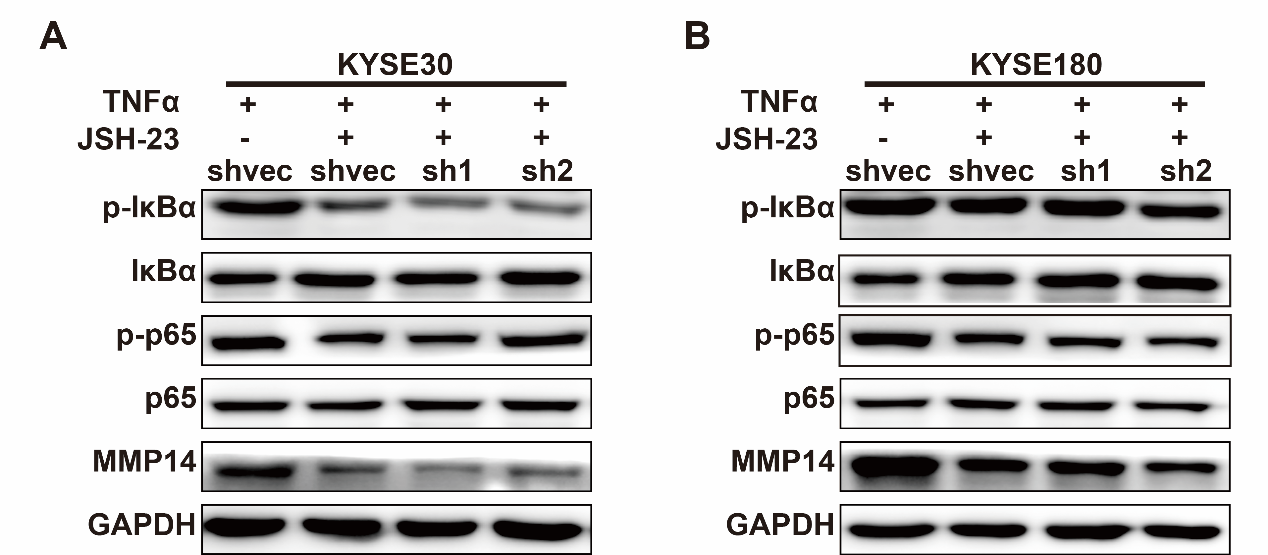
**Fig. S5 The NKILA-induced repression of MMP14 expression depended on NF-κB signaling.** NKILA-knockdown cells were incubated with TNFα with or without the NF-κB inhibitor JSH-23, and the expression levels of MMP14, p-IκB and p-p65 were measured by western blot. GAPDH served as the loading control.

**
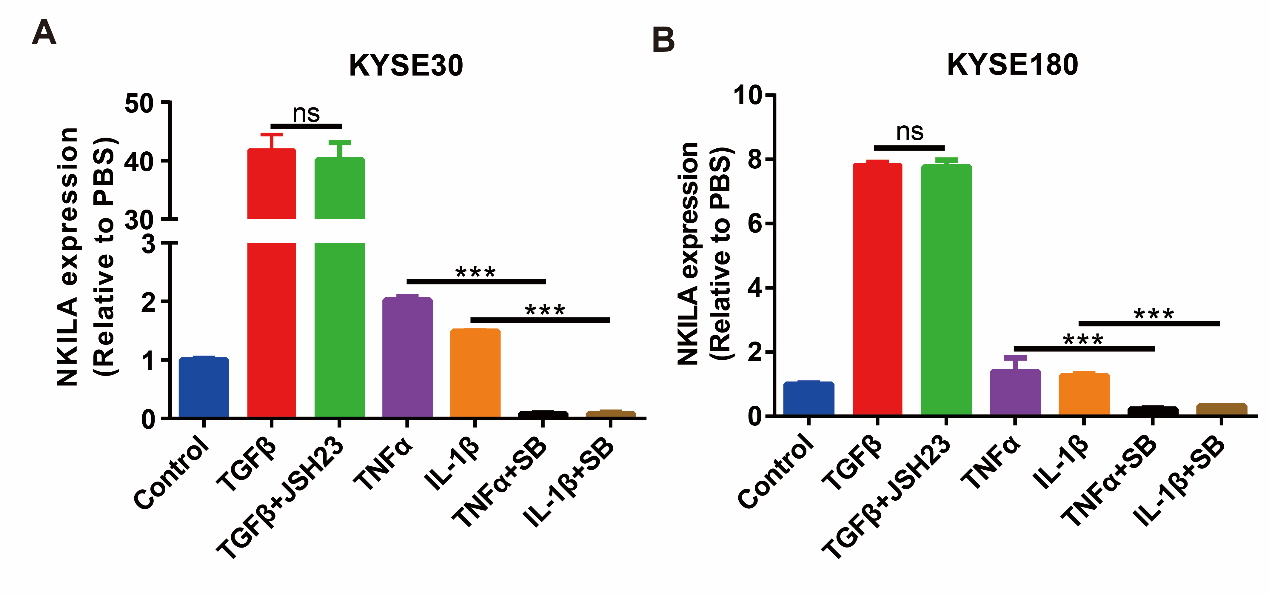
**

**Fig. S6** **TGF-β was the main signaling pathway to regulate NKILA expression level in ESCC**. The TGF-β pathway was the main signaling pathway that regulated NKILA expression levels in ESCC. NKILA expression levels were detected by qRT-PCR in KYSE30 (A) and KYSE180 (B) cells treated with TGF-β1 with or without JSH-23 and in ESCC cells treated with TNFα or IL-1β with or without SB505124 (SB). Data are presented as the mean ± SD, n = 3. Two-tailed Student’s t-test; *** p < 0.001; ns, no significant difference.

**Methods and materials for RNA-Seq**

**RNA quantification and qualification**

RNA degradation and contamination was monitored on 1% agarose gels. RNA purity was checked using the NanoPhotometer® spectrophotometer (IMPLEN, CA, USA). RNA concentration was measured using Qubit® RNA Assay Kit in Qubit® 2.0 Flurometer (Life Technologies, CA, USA). RNA integrity was assessed using the RNA Nano 6000 Assay Kit of the Bioanalyzer 2100 system (Agilent Technologies, CA, USA).

**Library preparation for RNA sequencing**

A total amount of 3 μg RNA per sample was used as input material for the RNA sample preparations. Firstly, ribosomal RNA was removed by Epicentre Ribo-zero™ rRNA Removal Kit (Epicentre, USA), and rRNA free residue was cleaned up by ethanol precipitation. Subsequently, sequencing libraries were generated using the rRNA-depleted RNA by NEBNext® Ultra™ Directional RNA Library Prep Kit for Illumina® (NEB, USA) following manufacturer’s recommendations. Briefly, fragmentation was carried out using divalent cations under elevated temperature in NEBNext First Strand Synthesis Reaction Buffer（5X）. First strand cDNA was synthesized using random hexamer primer and M-MuLV Reverse Transcriptase（RNaseH-）. Second strand cDNA synthesis was subsequently performed using DNA Polymerase I and RNase H. In the reaction buffer, dNTPs with dTTP were replaced by dUTP. Remaining overhangs were converted into blunt ends via exonuclease/polymerase activities. After adenylation of 3’ ends of DNA fragments, NEBNext Adaptor with hairpin loop structure were ligated to prepare for hybridization. In order to select cDNA fragments of preferentially 150~200 bp in length, the library fragments were purified with AMPure XP system (Beckman Coulter, Beverly, USA). Then 3 μl USER Enzyme (NEB，USA) was used with size-selected, adaptor-ligated cDNA at 37°C for 15 min followed by 5 min at 95°C before PCR. Then PCR was performed with Phusion High-Fidelity DNA polymerase, Universal PCR primers and Index (X) Primer. At last, products were purified (AMPure XP system) and library quality was assessed on the Agilent Bioanalyzer 2100 system.

**Clustering and sequencing**

The clustering of the index-coded samples was performed on a cBot Cluster Generation System using TruSeq PE Cluster Kit v3-cBot-HS (Illumia) according to the manufacturer’s instructions. After cluster generation, the libraries were sequenced on an Illumina Hiseq 4000 platform and 100 bp paired-end reads were generated.
